# Beyond Medical Therapy—An Update on Heart Failure Devices

**DOI:** 10.3390/jcdd11070187

**Published:** 2024-06-23

**Authors:** Luigi Falco, Fabio Valente, Aldo De Falco, Raffaele Barbato, Luigi Marotta, Davide Soviero, Luigi Mauro Cantiello, Carla Contaldi, Benedetta Brescia, Enrico Coscioni, Giuseppe Pacileo, Daniele Masarone

**Affiliations:** 1 Heart Failure Unit, Department of Cardiology, AORN dei Colli-Monaldi Hospital, 80131 Naples, Italy; luigifalco94@libero.it (L.F.); dr.valentefabio@gmail.com (F.V.); aldodefalco@yahoo.it (A.D.F.); raffaele.barbato96@gmail.com (R.B.); marotta.luigi.lm@gmail.com (L.M.); davide.soviero97@gmail.com (D.S.); luigicantiello3@gmail.com (L.M.C.); contaldi.carla@gmail.com (C.C.); gpacileo58@gmail.com (G.P.); 2 Department of Advanced Biomedical Sciences, University of Naples “Federico II”, 80131 Naples, Italy; bresciabenedetta@libero.it; 3 Cardiac Surgery Division, AOU San Leonardo, 84100 Salerno, Italy; coscionienrico@gmail.com

**Keywords:** heart failure, devices, teer, ccm, atrial shunt

## Abstract

Heart failure (HF) is a complex and progressive disease marked by substantial morbidity and mortality rates, frequent episodes of decompensation, and a reduced quality of life (QoL), with severe financial burden on healthcare systems. In recent years, several large-scale randomized clinical trials (RCTs) have widely expanded the therapeutic armamentarium, underlining additional benefits and the feasibility of rapid titration regimens. This notwithstanding, mortality is not declining, and hospitalizations are constantly increasing. It is widely acknowledged that even with guideline-directed medical therapy (GDMT) on board, HF patients have a prohibitive residual risk, which highlights the need for innovative treatment options. In this scenario, groundbreaking devices targeting valvular, structural, and autonomic abnormalities have become crucial tools in HF management. This has led to a full-fledged translational boost with several novel devices in development. Thus, the aim of this review is to provide an update on both approved and investigated devices.

## 1. Introduction

**Figure 1:**
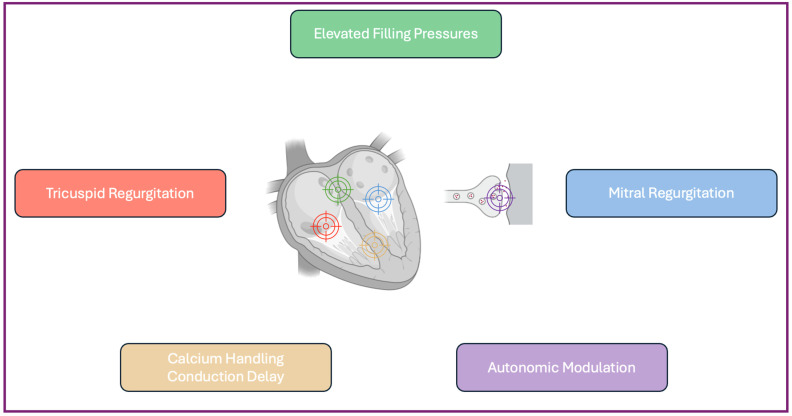
Pathways targeted by novel HF devices.

Historically, the mainstay of HF therapy has been represented by neurohormonal antagonists targeting different steps of the detrimental hyperactivation of the renin angiotensin aldosterone axis and sympathetic system, while diuretics have addressed congestion and relieved symptoms. In the last decade, novel drugs addressing different molecular pathways have emerged. Sacubitril/Valsartan further reduced mortality and HF-related hospitalizations compared to Enalapril in the seminal PARADIGM-HF (this study will evaluate the efficacy and safety of LCZ696 compared to Enalapril on the morbidity and mortality of patients with chronic heart failure) trial [1]. Sodium-glucose cotransporter-2 inhibitors (SGLT2i), especially Dapagliflozin and Empagliflozin, have gone from promising oral antidiabetic drugs with positive effects on weight to milestones in HF therapy, leading to the concept of quadruple therapy. This latter drug has been embraced by both European and American HF guidelines [2,3]. Additionally, based on clinical profiles [4], further medications are recommended to improve patients’ QoL and functional capacity and reduce hospitalizations, with Vericiguat being the first to enhance an endogenous pathway rather than inhibit it [5]. Despite these remarkable advances, the registry data are not reassuring. First, HF’s prevalence is rising worldwide, and not only because of population aging. Indeed, the risk factors burden is increasing, with more than half of the US adult population being at risk of HF or having pre-HF (stage A and stage B, respectively) [6]. Despite the availability of novel disease-modifying therapies [7], mortality has not declined since early 2010s, highlighting the high residual risk of HF patients and the dismal prognosis of the disease [8]. In addition, unlike HF with a reduced ejection fraction (HFrEF), the therapeutic options for HF with a preserved ejection fraction (HFpEF) or acute worsening events across the entire ejection fraction (EF) spectrum remain poor. This scenario has spurred the extensive development of technological approaches, broadening the landscape of HF devices. Some of them target valvular, structural, and electrophysiological abnormalities, have been extensively studied, and are recommended by guidelines. Many others are currently under investigation, targeting pathways not yet addressed by medical therapies (Figure 1). 

## 2. Mitral Valve Devices

Mitral regurgitation (MR) is classified as “primary” or “degenerative” when there is a disorder of the leaflets or sub-valvular apparatus and “secondary” or “functional” when the dysfunction is caused by an enlargement of the left ventricle or, less frequently, by an enlargement of the left atrium or annulus [9]. HF and MR are closely intertwined: left ventricular dilatation may cause leaflets tethering and a failure to coapt. However, severe MR worsens heart failure signs, symptoms, and quality of life (QoL) and increases hospitalizations and mortality compared to patients with mild or no MR [10]. Therefore, the optimization of guideline-directed medical therapy (GDMT) and CRT implantation when indicated should be the initial treatment. International guidelines recommend the use of transcatheter edge-to-edge repair (TEER) (class IIa) in patients with reduced a left ventricular ejection fraction, symptoms, and at least moderate to severe MR despite the GDMT if they have a high surgical risk, favorable anatomy, and no need for further cardiac surgery [9]. Two main systems are used for m-TEER: MitraClip (Abbot) and PASCAL (Edwards lifescience) (Table 1). In 2018, two RCTs evaluated the safety and efficacy of the Mitraclip compared to GDMT alone: MITRA-FR [11] and COAPT [12]. However, the results were conflicting. The MITRA-FR (Multicentre Study of Percutaneous Mitral Valve Repair MitraClip Device in Patients with Severe Secondary Mitral Regurgitation) study enrolled 304 patients with symptomatic heart failure (NYHA II−IV), LVEF 15–40%, severe MR, and at least one hospitalization for heart failure (HHF) in the last year. However, the primary endpoint (a composite of all-cause death or unplanned HF-related hospitalizations at 12 months) was not reached. Indeed, patients randomized to the interventional group did not show a reduction in all-cause mortality or their HFH rates at 12 months compared to those allocated to the GDMT group (54.6% vs. 51.3%, HR: 1.16, 95% CI 0.73–1.84), which was confirmed at a 24-month follow-up (63.8% vs. 67.1%, HR: 1.01, 95% CI 0.77–1.34) [13]. On the other hand, the COAPT (Cardiovascular Outcomes Assessment of the MitraClip Percutaneous Therapy for Heart Failure Patients with Functional Mitral Regurgitation) trial enrolled 614 patients with symptomatic heart failure, LVEF 20–50%, moderate or severe MR, a telediastolic diameter less than 70 mm, and at least one HHF or an BNP in serum increase. In this group of patients, a Mitraclip implantation on top of GDMT demonstrated a significant reduction in HHF (35.8% vs. 67.9%, HR 0.53, 95% CI 0.40–0.70) and a reduction in all-cause mortality compared to GDMT alone (19.1% vs. 23.2%, HR 0.62; 95% CI 0.46–0.82) after 24 months of follow-up [12]. The different subsets of patients enrolled may explain the divergent results of these studies [14,15]. COAPT enrolled patients with more severe MR (EROA 41 ± 15 mm^2^ vs. 31 ± 10 mm^2^) and minor ventricular dilatation (their mean indexed LV end-diastolic volume was 101 ± 34 mL/m^2^ vs. 135 ± 35 mL/m^2^) than MITRA-FR. The analysis of these data shows that MITRA-FR patients had an MR proportional to their ventricular dilatation, whereas COAPT patients had a non-proportional MR, which was often dependent on abnormal ventricular wall motion and had an eccentric jet, characterized as secondary MR, similar in mechanism to ‘primary’ MR. Differentiating between these two phenotypes allows us to distinguish the elements characterizing the prognosis of the two MRs. In “proportional” phenotypes, prognosis depends mainly on the ventricular dilatation and not on the grading of the MR, which is the opposite of disproportionate phenotypes [14,15]. In order to differentiate between the two phenotypes and thus choose the most suitable therapies, it may be useful to use the regurgitant volume/LVEDV ratio. Interestingly, the up-titration of therapy was not monitored in MITRA-FR and may not have been optimized prior to the intervention, while COAPT required maximum up-titration [16]. Furthermore, the use of sacubitril/valsartan in both RCTs was low. Finally, the interventional technique could be improved in COAPT. There was a greater use of clips, with fewer complications and a greater efficacy, in COAPT than in MITRA FR, evidenced by the at least moderate to severe MR being lower both acutely (5% vs. 9%) and at 12 months (5% vs. 17%) [16]. The recently published 3- and 5-year follow-ups of COAPT confirmed the positive results previously achieved [17,18]. Both HHF and all-cause mortality were decreased (HR 0.53, 95% CI 0.41–0.68 and HR 0.72, 95% CI 0.58–0.89, respectively). The effect size, however, was reduced, probably due to the crossover of patients from the control group to the device group, allowed after 24 months of follow-up. Device-related safety events occurred only in four patients (1.4%). All these events, though, occurred within the first 30 days after surgery. The PASCAL Transcatheter Valve Repair System was approved in 2019 for the treatment of functional, degenerative, or mixed MR in standard or difficult anatomy since it was shown to be safe and effective in the CLASP (The CLASP Study Edwards PASCAL TrAnScatheter Mitral Valve RePair System Study) study, reducing the grading of MR from at least moderate–severe to only moderate or mild at a one-month follow-up (98% and 86%, respectively) [19]. The 1-year follow-up of CLASP showed an improvement in QoL and functional capacity, with a sustained reduction in the grading of regurgitation [20]. Consistent findings were recognized also in patients with complex valvular anatomy [21]. In addition, a sub-analysis of the study, which included only functional etiologies, showed that the reduction in the degree of regurgitation and improvement in NYHA class were even greater than with COAPT [22]. The non-inferiority of the PASCAL system to the Mitraclip system was recently assessed in the CLASP IIF (NCT03706833) study [23]. Enrolled patients were deemed to be at prohibitive surgical risk but suitable for mitral TEER. The primary safety and efficacy endpoints were met (major adverse events rate and sustained MR < 2+, respectively). Moreover, at six months, fewer patients had a residual MR > 2+ (14% vs. 26.9%). In recent years, new therapeutic perspectives other than edge-to-edge techniques have been developed, such as mitral annuloplasty devices, which can be classified as direct (Cardioband Mitral System, Mitralign Percutaneous Annuloplasty System) or indirect (Carillon Mitral Countour System). The purpose of such devices is to improve leaflets’ coaptation by reducing the annulus diameter in secondary MR. A single-arm prospective multicenter study (NCT01841554) evaluated the feasibility and safety of the Cardioband in 60 patients on GDMT with functional and at least moderate MR [24]. A 1-year follow up was possible for 39 patients and 70% of them had mild MR. The rates of MR reduction were comparable to those of Mitraclip [24]. Mitralign is a direct annuloplasty system placed retrogradely through the aorta which has been shown to reduce MR in 50% of patients and improve their NYHA class and 6-MWT (Mitralign Percutaneous Annuloplasty First in Man Study, NCT01852149) [25]. The Carillon Mitral Countour System has shown, in the REDUCE FMR (NCT02325830) study, reverse remodeling and improved left ventricular volumes, regurgitation grading, and NYHA class [26]. However, the effects were modest and implant success rates lower compared to direct annuloplasty techniques. This notwithstanding, a recent pooled analysis including patients regardless of MR type reported consistent benefits for both proportionate and disproportionate MR, potentially breaking the emergent paradigm that patients with proportionate MR should receive therapies targeting the left ventricle rather than the mitral valve [27]. Tendyne is a self-expanding porcine tri-leaflet valve positioned transapically and anchored to the apex of the left ventricle, used for secondary or mixed MR of at least moderate–severe grade in patients at high or prohibitive surgical risk. A non-randomized study showed a high surgical success rate (96%), with improvement in NYHA classes and HHF rates [28]. A comparison study (i.e., SUMMIT, NCT03433274) between mTEER and Tendyne in patients with moderate-to-severe MR or symptomatic mitral valve disease due to severe mitral annular calcification (MAC) is ongoing. Its preliminary findings suggested that Tendyne was associated with greater improvements in MR reduction and NYHA class compared to TEER, despite early drawbacks in survival. TEER now represents a revolution in the correction of MR, even if the echocardiographic criteria for selecting the most appropriate patient and the intervention times are still uncertain. New trials are needed to make transcatheter treatment tailored to MR phenotypes, and new devices are being tested to ensure better success in the next few years.

## 3. Tricuspid Valve Devices

Tricuspid regurgitation (TR) is frequently found in the general population. Almost 70% of patients present mild regurgitation, which does not assume pathological significance [30]. Moderate or severe regurgitation can be linked to a primary lesion of the valve itself, congenital or acquired, or, much more frequently, to left-sided heart dysfunction, pulmonary hypertension, or the implantation of cardiac implantable electronic devices (CIEDs). The latter has recently been recognized as an increasing cause of TR, since the leads’ positioning could damage different elements of the apparatus. Furthermore, the risk of infection and detrimental effects of conventional right ventricular pacing increase the risk TR progression. Either a volume or pressure overload of the right ventricle causes a progressive dilation of the tricuspid annulus, resulting in failed leaflet coaptation and overt regurgitation [31], which significantly worsen the patient’s HF prognosis [29]. Recently, the interest in TR treatment has increasingly grown. Until a few years ago, the only therapeutic options were diuretics to relieve symptoms and, in carefully selected cases, by surgical valve repair or replacement. According to the 2021 ESC guidelines, surgery is recommended in patients with severe TR either undergoing left-sided valve surgery or with symptoms and a dilated right ventricle (class IC and class IB, respectively), to promote the remodeling of the right ventricle and improve its function [9]. Moreover, surgical repair is preferable to replacement, which should be considered only if the leaflets are tethered and the annulus is severely dilated. However, data are scant, and the correct surgical timing is not well defined. Additionally, its high surgical risk and the success of percutaneous transcatheter interventions for both the aortic and mitral valve have paved the way for transcatheter tricuspid valve replacements (TTVrs) (class IIb), especially in patients without right ventricular dysfunction and/or pulmonary hypertension. Several devices are available today (Table 2).

Based on their mechanism of action, they can be divided into coaptation and leaflets devices, annuloplasty devices, the heterotopic implantation of devices into the caval site, and orthotopic transcatheter tricuspid replacement. Coaptation devices include the Mitraclip/Triclip, Forma, and Pascal. The successful MR reduction achieved through Mitraclip implantation led to its off-label use for the tricuspid valve. It corrects regurgitation by tying the anterior or posterior leaflet to the septal leaflet [39,40]. Recently, the Triclip device has been developed. It was manufactured to better adapt to the more complex anatomy of the tricuspid. The TRI-LUMINATE study (TRILUMINATE Study With Abbott Transcatheter Clip Repair System in Patients With Moderate or Greater TR, NCT03227757) enrolled 85 symptomatic patients with moderate or severe TR undergoing Triclip implantation and demonstrated a reduction in TR by at least 1 degree at 30 days in 86% of cases. Major adverse events occurred in only 6% of patients, which was much less than expected, corroborating the safety of the procedure [41]. Recently, the pivotal TRILUMINATE trial (NCT03904147) studied, in a randomized fashion, the impact of the Triclip compared to GDMT [32]. The primary endpoint (a hierarchical composite including all-cause death or tricuspid valve surgery, HF-related hospitalizations, and improved QoL assessed by the KCCQ questionnaire) was reached (win ratio 1.48; 95% CI, 1.06–2.13; *p* = 0.02). However, these findings were essentially driven by improvements in QoL, which were strictly related to the degree of TR reduction. Indeed, the study did not reveal any differences in mortality or HF hospitalization rates within a one-year period. The not-very-high-risk population enrolled and the limited follow-up may have been responsible for the lack of benefit in terms of harder outcomes. Moreover, while it is important to acknowledge the potential for bias due to the open-label methodology used, the discovery of an enhanced QoL aligns with earlier, non-randomized investigations of tricuspid TEER [42]. The Forma system uses a foam-filled spacer inside the regurgitant orifice and a track that is anchored to the right ventricle, onto which the spacer is released. The spacer increases the coaptation surface of the tricuspid leaflets and thus reduces regurgitation. A recent trial included 19 patients who underwent TTVr with Forma. Among the 15 successfully implanted patients with at least a 24-month follow-up, persistent improvements in NYHA functional class and TR degree were observed [43]. The Pascal system combines Mitraclip and Forma techniques: a spacer in the center of the regurgitant orifice is tied to the leaflets by two clips. The CLASP TR EFS (Edwards PASCAL TrAnScatheter Valve RePair System in Tricuspid Regurgitation [CLASP TR] Early Feasibility Study, NCT03745313) study enrolled 34 patients, 29 of whom received the implant. In 85% of cases there was a reduction in TR of at least 1 degree at 30 days [33,44]. The devices available for annuloplasty are divided into ring devices (Cardioband, Edwards Life-sciences, Irvine, California; Iris, Millipede, Inc., Santa Rosa, CA, USA; Traipta, National Institutes of Health and Cook Medical, Bloomington, Indiana) and suture-based devices (Trialign, Mitralign, Inc., Boston, MA, USA; Tricinch, 4Tech Cardio Ltd, Galway, Ireland; Pasta; Mia, Micro Interventional Devices, Inc., Newton, PA, USA; DaVingi, Cardiac Implants, Wilmington, Delaware). Among the ring devices, the most used is the Cardioband. It is an adjustable ring that is anchored to the tricuspid annulus with screws. Once completely positioned, it is contracted to reduce TR. The TRI-REPAIR (TrIcuspid Regurgitation RePAIr With CaRdioband Transcatheter System, NCT02981953) was a single-arm, prospective, multicenter study that enrolled 30 patients with inoperable TR. Six months after Cardioband implantation (100% success rate), a significant reduction in TR and improvements in QoL and exercise capacity were achieved [34]. These results were maintained over time, as shown in the 2-year follow-up report [45]. More recently, patients with severe functional TR despite medical therapy were recruited in the US multicenter Edwards Cardioband Tricuspid Valve Reconstruction System Early Feasibility study (NCT03382457). At 1 year, 73% of patients reached at least moderate TR, whereas nearly all patients experienced improvements in their QoL. Moreover, their echocardiographic parameters continued to reduce compared to baseline throughout the follow-up, suggesting reverse remodeling [46]. Suture annuloplasty devices, such as Trialign and Tricinch, use suture techniques that resemble surgical ones. Trialign reduces the diameter of the annulus through the plication and subsequent bicuspidation of the tricuspid valve through the obliteration of the posterior leaflet. Tricinch also reduces the diameter through a corkscrew anchor positioned in the annulus and a band fixed to a self-expanding stent in the inferior vena cava, which puts in tension the anchor. The multicenter RCT SCOUT (Percutaneous Tricuspid Valve Annuloplasty System for Symptomatic Chronic Functional Tricuspid Regurgitation, NCT02574650) demonstrated an 80% success rate for Trialign with a 30-day significant echocardiographic reduction in tricuspid annulus diameter and effective regurgitant orifice area (EROA) in the 15 patients in whom it was implanted [35]. The PREVENT (Transcatheter Treatment of Tricuspid Valve Regurgitation with the TriCinch System™, NCT02098200) study evaluated the safety and efficacy of the TriCinch system. Among the 24 patients treated, the procedure was successful in 18 of them (85%), with a significant reduction in TR. However, late complications highlighted the need for structural and technical improvements [47]. Finally, heterotopic bioprostheses have been designed to reduce the systemic venous congestion resulting from TR in the vena cava. Among these are the TricValve, which is made up of two self-expanding biological valve prostheses implanted in the caval site, and the Tricento, a self-expanding valved stent bioprosthesis spanning from IVC to SVC [36,37]. As an alternative to heterotopic implantation, a transcatheter valve replacement can be used. NaviGate, LuxValve, Trisol, and Evoque are just some of the bioprostheses available. Large-scale trials to evaluate the efficacy and safety of heterotopic/orthotopic devices are still ongoing. Recently, data from the multicenter registry TRISCEND evaluating the safety and efficacy of the Evoque system have been published [38]. A total of 176 patients, mostly female, with at least severe TR and a NYHA class ≥ III were enrolled. After 1 year, 97,6% of patients had at least mild TR, showing the system’s greater efficacy in decreasing regurgitation compared to TEER. On the other hand, 30-day major adverse events were reported in 18.6% of the cohort and 13.3% of patients required permanent pacemaker implantation due to heart block [38]. Therefore, the difficult anatomy of the valve, its close relationship with the conduction system, and the high risk of thrombosis due to the low pressure of the right-sided heart circulation make the use of bioprostheses challenging. In conclusion, the tricuspid valve seems to be no longer the forgotten valve for which limited therapeutic options and prohibitive surgical risk prevented interventional approaches. The emerging safety data and the encouraging efficacy results in terms of TR degree reduction, QoL, and functional capacity improvements are paving the way for a new era in the treatment of a common yet ominous valvular diseases. Nevertheless, further studies are needed to guide interventional cardiologists through the evolving landscape of devices. Despite the variety, choosing the correct device for each patient remains difficult.

## 4. Interatrial Shunting

HFpEF, in recent years, has become a hot topic due to its increasing prevalence and the shortage of pharmacological therapeutic treatments, except for SGLT2i. This is why attention has turned toward the use of devices that can counteract the pathophysiological pathways that impair QoL and cause exercise intolerance. Indeed, increased left ventricular and left atrial filling pressures determine pulmonary congestion, provoking the most common symptom, i.e., dyspnea at exertion [48]. This is due to the passive retrograde transmission of pressure through the pulmonary veins to the venous capillaries, leading to the possibility of pulmonary edema, an increased V/Q mismatching causing increased ventilator demand and a declination of right ventricular performance because of increased right ventricular afterload [48,49,50]. Maeder et al. [51] have reported an inverse relationship between pulmonary artery wedge pressure (PAWP) and both workload and peak VO2, establishing increased filling pressures as hemodynamic determinants of poor functional capacity. These finding are consistent with a previous study which showed that a higher PAWP, albeit normal, at rest and a PAWP > 25 mmHg compromised cardiac output increases during exercise [52]. Additionally, the established prognostic significance of PAWP has made it an appealing target for innovative interventions. Hence, several percutaneous techniques to reduce left atrial pressure, creating an iatrogenic left-to-right interatrial shunt, have been employed and many other are under development (Table 3). Most of the studies evaluated the same type of device, i.e., the Corvia interatrial shunt device (IASD) (Corvia Medical). The first feasibility pilot study by Sondegaard et al. [53] in HFpEF patients suggested early benefits consisting of a reduced PAWP at rest and 1-year improvements in both 6MWD and Minnesota Living with Heart Failure (MLWHF) scores. The REDUCE LAP-HF (A Study to Evaluate the DC Devices, Inc. IASD™ System II, Tewkesbury, Massachusetts, to REDUCE Elevated Left Atrial Pressure in Patients With Heart Failure, NCT01913613), an open-label, single-arm, phase 1 study, assessed the impact of device implantation on both at-rest and exertional hemodynamics. Sixty-eight symptomatic patients, despite pharmacological therapy, were recruited. The eligibility criteria included being > 40 years of age, an LVEF > 40%, and elevated LAP, with a resting PAWP > 15 mmHg or a PAWP > 25 mmHg during exercise [54]. No device safety issues were reported, patency at six months was assured, and more than half of the patients had a reduction in their PAWP, as well as an increase in their cardiac output. In addition, improvements in MLWHF, 6-MWT, and functional NYHA class followed these hemodynamic changes. A subsequent analysis provided a deep dive into the pathophysiology of interatrial shunting. Indeed, it showed that the patients who were most likely to benefit from the device were those with a wider baseline gradient between their PAWP and CVP, regardless EF (range explored: 40–49% vs. >50%). Moreover, among the patients who accepted an extended follow-up (12 months), these hemodynamic effects were sustained [55]. The REDUCE-LAP HF 1 (Reduce Elevated Left Atrial Pressure in Patients With Heart Failure, NCT02600234) was a phase 2, randomized, parallel-group, sham-controlled, multicenter study aimed to assess exercise PAWP one month after the intervention [56]. Forty-four eligible patients with an EF > 40% and exercise PAWP > 25 mmHg were randomly allocated (1:1) to an IASD implantation or a sham procedure. After 1 month, the treatment group experienced a significant reduction in PAWP, measured both after exercise and after a passive leg raise (*p* = 0.028 and *p* = 0.024, respectively). After 12 months, the incidence of cardiac, cerebrovascular, and renal events was not different between the two groups. Additionally, although the study was not powered to assess clinical outcomes as exploratory endpoint, a trend toward fewer HF-related hospitalizations emerged in the IASD group. Further evidence supporting the beneficial effects of the IASD comes from a pooled analysis of the first two REDUCE-LAP HF studies [57]. Despite an augmented pulmonary flow, the increased oxygen content in the pulmonary artery improved pulmonary vascular resistance. Interestingly, these changes were even more pronounced in patients with atrial fibrillation. The consistent findings across the studies paved the way for a large RCT focusing on hard endpoints. The REDUCE LAP-HF II (NCT03088033) was a phase 3, randomized, blinded, international study of 626 patients, including a sham control group, with an elevated resting or exercise PAWP and a left ventricular ejection fraction > 40% [58]. Despite its rigorous methodology, the primary endpoint—a hierarchical composite of cardiovascular death or non-fatal ischemic stroke at 12 months, the total HF events (including both hospital admissions and urgent outpatient visits requiring the up-titration of oral diuretic therapy or a switch to intravenous diuretics) at 24 months, and the change in health status at 12 months—was not met (win ratio 1.0, 95% CI 0.8–1.2]; *p* = 0.85). Interestingly, a prespecified subgroup analysis showed significant interactions. Male sex, a right atrial volume index > 29.7 mL/mq, and a PA systolic pressure at 20 W of exercise (>70 mmHg) favored the sham control. These findings led the authors to conduct a post hoc exploratory analysis, which demonstrated the significant benefits in patients without latent pulmonary vascular disease (PVR < 1.74 WU) (win ratio 1.28, *p* = 0.032). Despite an apparent negative RCT, it could be helpful in further tailoring the phenotype of patients responsive to IASD. Thus, it is important for future investigations to keep in mind these findings when defining their inclusion criteria. From this finding, an analysis by Borlaug et al. [59] was conducted to evaluate exercise hemodynamics and latent pulmonary vascular disease (defined by an exercise PVR ≥ 1.74 WU). Patients with PVD had more elements that suggest a worst response to an IASD therapy, such as a lower TAPSE, higher RA volumes, higher right atrial pressure, and lower CO and peak exercise levels. Latent PVD has been hypothesized to be a predisposing factor for an inability to sustain increased pulmonary flow. The NoYa is a system consisting of a self-expanded flowerlike nitinol stent fixed within a delivery sheath connected to a radiofrequency generator. After ablation, the device, which can adjust the size of the defect that is created, is removed. After preliminary data, this technique was considered feasible and safe for subjects with HFrEF and HFmEF [60]. The RAISE II (Radiofrequency Ablation-Based Interatrial Shunt for Heart Failure, NCT05375110) open-label study is ongoing, evaluating the impact of the procedure on cardiovascular mortality and HF-related hospitalizations in patients with an LVEF > 15% and PAWP at rest >15 mmHg. The ALT-FLOW study evaluated a different path to reduce left atrial filling pressure [61]. The Edwards left atrial to coronary sinus APTURE Transcatheter Shunt System has been evaluated in 87 symptomatic patients with HF. Device implantation was successful in 79 patients. After 6 months, both exercise PAWP and functional status significantly improved compared to baseline (*p* < 0.001). Hemodynamic beneficial effects were seen both in cohort A (PVR < 3 WU) and cohort B (PVR > 3 WU). The V-wave Ventura shunt system consists of a nitinol frame covered with ePTFE, which creates a smaller shunt (5 mm) compared to other devices. The first design of the device included a one-way valve to avoid a right-to-left shunt. Despite significant improvements in both NT-proBNP levels and NYHA class, patients developed shunt stenosis [62]. The modified device has been evaluated in the RELIEVE-HF (Reducing Lung Congestion Symptoms in Advanced Heart Failure, NCT03499236), a randomized, sham-controlled study, including in HF patients regardless their LVEF and according to solely their rest hemodynamics [63,64]. There was no difference in the primary effectiveness endpoint, a composite of all-cause mortality, left ventricular assist device (LVAD)/heart transplantation, HF-related hospitalizations, outpatient worsening HF events, and changes in KCCQ. However, the subgroup of patients with reduced LVEFs had lower HF hospitalization rates. Interestingly, the V-wave device increased worsening events and mortality in HFpEF patients. Elevated left atrial pressure is critical in the pathophysiology of HFpEF and HFmEF, suggesting, as a possible therapeutic weapon, that the creation of communication at the interatrial level allows for decompression of the left sections, and thus less lung congestion. Although the data from the studies to date are not promising overall, the pathophysiological substrate is high, and there is still room, through new studies, to understand which device is best and determine whether certain subgroups or better patient selection will lead to different and more promising results.

## 5. Implantable Cardioverter Defibrillator

Sudden cardiac death (SCD) is an important cause of death in HF patients. Despite the advances in medical therapy, a significant residual risk persists [65]. Several RCTs have established the key role of transvenous implantable cardioverter defibrillators (TV-ICDs) in both primary and secondary SCD prevention [66]. However, significant rates of both early and long-term complications with TV-ICDs led to the development of subcutaneous ICD (s-ICD) devices. Similarly to TV-ICDs, s-ICDs consist of a pulse generator and a defibrillator lead. However, the entire system is extra-thoracic, avoiding interactions with the vasculature and heart. Patients at high risk for infection (i.e., previous TV-ICD infection, immunodeficiency, or on hemodialysis) or with limited vascular access, who do not have pacing needs, have a class I recommendation for s-ICD implantation. Plenty of studies have proved their efficacy and safety in both primary and secondary prevention settings. Most of them included patients with HFrEF with both ischemic and non-ischemic etiologies [67]. However, even the s-ICD has some drawbacks: (1) Besides pacing in the immediate period after shock, the device is unable to deliver either pause prevention pacing or anti-tachycardia pacing (ATP). Therefore, an s-ICD implantation is contraindicated in patients needing pacing. (2) Sensing limitations related to its extra-thoracic location undermine arrhythmia detection. Therefore, to limit far-field sensing issues, all patients must undergo screening prior to implantation. (3) Its defibrillation thresholds are higher than those of TV-ICDs and may be unstable over time. Indeed, adipose tissue has a high impedance, and an inadequate position of the generator can drastically reduce the amount of current crossing the myocardium. This technical issue has a direct impact on the hardware, translating into a larger battery and a reduced lifespan of the device. The result is an increased number of interventions over time, increasing procedure-related risks and costs. (4) Inappropriate shocks, which can be due to air entrapment in the generator pocket or T-wave oversensing. Among the limitations of s-ICD devices, the lack of pacing seems to be the most feared one. Strategies to overcome this issue are under evaluation. The MODULAR-ATP (Effectiveness of the EMPOWER Modular Pacing System and EMBLEM Subcutaneous ICD to Communicate Antitachycardia Pacing) is an ongoing prospective, non-randomized, multicenter study aiming to demonstrate the safety and efficacy of the modular cardiac rhythm management (mCRM) system [68]. It consists of a leadless pacemaker (EMPOWER) able to deliver either bradycardia pacing support or ATP when prompted by s-ICD sensing (EMBLEM). A different option that does not rely on intercommunicative devices is the extravascular ICD (EV-ICD), in which the lead is implanted in a substernal position, allowing direct contact with the RV. This guarantees lower defibrillation thresholds than the s-ICD alongside good pacing and sensing. Recently, the results of the Extravascular ICD Pivotal Study (NCT04060680) have been published [69]. The EV-ICD was effective in terminating ventricular arrhythmias. No major intraprocedural complications were reported. However, 9.7% of patients had inappropriate shocks, mostly due to P-wave oversensing. Larger studies with a longer follow-up are needed to define the role of these devices in SCD prevention. 

## 6. Cardiac Resynchronization Therapy

CRT has revolutionized the treatment of HFrEF since the early 1990s. Conduction abnormalities, including branch blocks, are often observed in patients with HF, altering the timing and organization of their ventricular contraction and exposing the insufficient heart to further mechanical disadvantages. As a result, suboptimal ventricular filling, reduced left ventricular contractility, a prolonged duration of mitral regurgitation, and paradoxical septal motion occur. Altogether, these abnormalities are known as “ventricular dyssynchrony”, evident on surface ECG as a wide QRS interval (>120 ms). This represents the surrogate marker of ventricular dyssynchrony used to select HFrEF subpopulations in previous RCTs. Ventricular dyssynchrony affects nearly one-third of HFrEF patients and is associated with higher morbidity, mortality, and SCD rates [70,71]. Since the early 2000s, many studies evaluating the role of CRT have been conducted. The MUSTIC RCT, a single-blind crossover study, was the first to demonstrate the efficacy of CRT in improving functional capacity and the QoL in patients with HFrEF, NYHA Class III/IV, and intraventricular conduction disorders [72]. These results were later confirmed by the MIRACLE study, which enrolled a larger population [73]. However, the two studies that gave definitive relevance to CRT, demonstrating its beneficial effects on hard endpoints such as mortality and hospitalizations, in patients with moderate–severe symptomatic HFrEF, were the COMPANION [74] and the CARE-HF RCTs [75]. The COMPANION was the first RCT demanding triple neurohormonal inhibitors as background therapy. In addition, while both RCTs showed a reduced incidence of primary endpoints in the experimental groups (0.63 (95% CI: 0.51 to 0.77) and 0.76 (95% CI: 0.63 to 0.90), CARE HF and COMPANION, respectively), only CARE-HF demonstrated a significant reduction in mortality with CRT-P (hazard ratio: 0.64; 95% CI: 0.48 to 0.85; *p* < 0.002). Subsequently, the MADIT-CRT, REVERSE, and RAFT trials were designed to investigate the efficacy of CRT in HF patients with a wide QRS complex and mild symptoms (NYHA class I–II) [76,77,78]. In these trials, patients were randomized to CRT-ON and CRT-OFF. Briefly, REVERSE showed significant reverse remodeling, MADIT-CRT showed lower hospitalization rates, and RAFT also showed a significant reduction in mortality in the CRT arm. Therefore, the benefits of CRT shown in these studies are consistent with those of older studies conducted in patients with more severe HF symptoms. A widely accepted sequela of right ventricular pacing in patients implanted with PM or ICD devices is LV systolic disfunction due to intraventricular dyssynchrony, worsening clinical outcomes [79]. In HFrEF patients with a significant right ventricular pacing burden (>20%), the upgrade to CRT-D compared to ICD therapy met the primary composite endpoint (all-cause mortality, HF hospitalizations, and a <15% reduction of left ventricular end systolic volume) (odds ratio 0.11; 95% CI 0.06–0.19; *p* < 0.001) in the BUDAPEST-CRT Upgrade study [80]. Patients randomized to the upgrade intervention also had a significantly lower risk of meeting the secondary endpoint, which included exclusively clinical outcomes (all-cause mortality and HF hospitalizations) (HR 0.27; 95% CI 0.16–0.47; *p* < 0.001), while device- or procedure-related events were not statistically different between the two groups. Despite the amount of data supporting beneficial effects of CRT, the CRT survey II showed that only about one-third of CRT candidates are implanted [81]. The complexity of the procedure and the existence of CRT non-responders seem to be key factors to physicians’ inertia [82]. Finally, the drawbacks with right ventricular pacing and the limitations of biventricular pacing (BiV) as a method to deliver CRT prompted the development of more physiological pacing options. A compelling rationale for conduction system pacing (CSP) is its ability to restore physiologic ventricular activation. While classic CRT achieves a reduction in LV dyssynchrony, CSP may result in the complete restoration of cardiac electrical depolarization and repolarization, further improving LV systolic and diastolic performance. Observational studies have demonstrated that His-bundle-pacing (HBP) or direct left-bundle-branch pacing can result in highly efficient resynchronization [83]. The His-SYNC [84] and His-alternative [85] RCTs compared HBP to BVP. HBP was at least equivalent to BVP in terms of its QRS narrowing and echocardiographic response. However, a high percentage of patients randomized to HBP required crossover to BVP. In the His-SYNC trial, the high crossover rate was attributed to the inclusion of patients with an intraventricular conduction delay (IVCD) who were not amenable to QRS correction and reverse remodeling, whereas, in the His-alternative trial, which excluded patients with an IVCD, crossovers were less frequent and overall procedural success was higher. Permanent transseptal Left Bundle Branch Area Pacing (LBBAP) is a promising new pacing method feasible as a primary technique for both bradyarrhythmia and heart failure indications. According to the Multicentre European Left Bundle Branch Area Pacing Outcomes Study (MELOS), LBBAP is feasible as a primary pacing strategy despite its more challenging procedure. Indeed, complications with the transseptal lead route are not rare and the learning curve is gradual [86]. However, concomitant distal conduction disorders undermine LBBAP’s ability to restore electrical synchrony. Hence, sequential “optimized” pacing strategies have emerged as a potential solution to address residual conduction delays. In a study conducted by Vijayaraman et al., 27 patients with advanced HF (NYHA ≥ III despite GDMT), who failed to achieve a reduction in their QRS duration with HBP, underwent additional LV pacing provided by a His-Optimized CRT (HOT-CRT) [87]. Both the clinical and echocardiographic response rates were >80%. Consistent findings were observed in patients with an IVCD and in “conventional CRT” non-responders. These results were confirmed in an elegant proof-of-principle study, in which Zweerink et al. demonstrated reductions in both LV and RV activation times [88]. A different approach was evaluated by Jastrzebski et al.; 112 non-consecutive patients (22% IVCD), for whom CRT was indicated, were enrolled in a prospective observational multicenter study [89]. All electrophysiologists involved in the study had LBBAP expertise and were encouraged to achieve LBB capture. LBBAP-optimized CRT (LOT-CRT) implantation was successful in 81% of patients. QRS narrowing was greater with LOT-CRT than BiV-CRT or LBBAP alone. In addition, the EF, LV volumes, and NYHA class improved while NT-proBNP levels were halved. These results were even greater in patients with a successful LBB capture. Since, among patients with an indication for CRT implantation, those with an IVCD have poor responses and worse outcomes, Chen et al. conducted a non-randomized study focusing on this subgroup. Eighty-three patients were assigned to LOT-CRT or BiV-CRT [90]. The LOT-CRT group had significantly greater improvements in their clinical, echocardiographic, and laboratory measurements throughout the study (24 months). Finally, in the LOT-CRT group, the primary endpoint, a composite including mortality and HHF, was significantly reduced (HR 0.33, CI 0.14–0.77; *p* = 0.035), paving the way for larger studies evaluating hard endpoints. Finally, it is worth mentioning the wireless WiSE-CRT system. It includes a transmitter, located subcutaneously above an intercostal space, that detects the RV pacing pulse originating from the coimplanted device and generates an ultrasound signal. This latter is received by a transmitter implanted in the LV wall and converted into electrical energy to provide biventricular pacing [91]. Currently, the device is approved for three indications that resemble the three group of patients studied in the prior WISE-CRT study: patients with an indication for CRT implantation or CRT upgrade in whom a coronary sinus LV lead placement failed and patients non-responsive to conventional CRT. Both the SELECT-LV study and the WiCS-LV Post Market Surveillance Registry reported consistent benefits in terms of EF and QRS duration; however, procedure-related adverse events remain an issue [92,93]. Finally, the SOLVE-CRT trial demonstrated reverse remodeling six months after randomization [94]. Despite the advent and growing interest in new cardiac resynchronization techniques, to date the CRT achieved by biventricular pacing remains the only technique to have a class I recommendation in current guidelines.

## 7. Cardiac Contractility Modulation

Cardiac contractility modulation (CCM) is a relatively recent therapeutic option for patients suffering from heart failure. It stems from experimental evidence in vitro, which showed that applying a positive amplitude current to myocardial cells during the absolute refractory period can have a positive inotropic effect [95]. Early feasibility studies demonstrated that the delivery of the stimulus through an endovenous electrode, similarly to how conventional pacemaker therapy is carried out, could swiftly improve cardiac contractility, with a statistically significant increase in pressure generated by the left ventricle [96]. At the time of writing this review, there is only one device available that can deliver CCM therapy: the OPTIMIZER system (Impulse Dynamics). The device consists of a generator with a wirelessly rechargeable battery and two or three active fixation cardiac electrodes. Two of the leads are connected to the right ventricular aspect of the interventricular septum and deliver the CCM impulse; the third is positioned in the right atrial appendage and serves to monitor cardiac activity to allow for the correct timing of the stimulatory impulses. The latest iteration of the OPTIMIZER device, the OPTIMIZER Smart, utilizes some updated sensing algorithms that allow it to function without an atrial lead [97]. The exact nature of CCM’s effect on the heart is still being investigated. It is believed that its action happens through a variety of different mechanisms, but the main one is related to cellular calcium trafficking. In the failing myocardium, L-type calcium channels are less active [98] and Sarco-Endoplasmic Reticulum Calcium ATPase (SERCA) activity is reduced. This results in a less pronounced calcium spike during systole and a delayed reuptake of calcium into the sarcoplasmic reticulum during diastole, thus contributing to reduced inotropism, defective relaxation, and an arrhythmic effect. CCM has been found to restore calcium kinetics in the failing heart. Furthermore, CCM can contribute to the positive remodeling of the myocardium, reverse the maladaptive fetal gene program, and help reduce cardiac fibrosis [99]. The first clinical trial to study the effects of CCM was the FIX-HF-4, which employed a double-blind, randomized, crossover design modelled after the MUSTIC trial of CRT. In this study, 164 HFrEF patients were selected. The inclusion criteria required that they be on stable, optimized medical therapy and have an ejection fraction of less than 35%. All the patients in the study received implantation of the OPTIMIZER device, but they were randomized to one of two groups. One group had the device activated right away, and turned off at 12 weeks, while the second group received sham therapy for 12 weeks, with the device being activated during the second phase. Of note, during the implantation procedure, the pressure differential generated by the left ventricle was monitored, and patients who did not achieve a 5% increment with stimulation were excluded from the study. This trial showed an increase in the primary endpoints of VO2 max and QoL according to the MLWHF questionnaire administered to both groups during the first phase, demonstrating a large placebo effect (Table 4). The patients of group 1, who were later switched to sham therapy, showed a decline in functional capacity and QoL, whereas those that switched from sham to active therapy showed an improvement in these metrics. The safety profile appeared to be equal during the two phases of the study for both groups [100]. Later, the FIX-HF-5 study enrolled 428 subjects with advanced heart failure (defined in this case as an LVEF less than 35% and NYHA class III or IV). The subjects were randomized to either OMT alone or OMT and CCM implantation. Due to the planned study duration of 12 months, it was felt to be unethical to implant an inactive device. Therefore, the study employed an unblinded design. The primary endpoint was an improvement in the ventilatory anaerobic threshold, which was not reached; however, the treatment group did show statistically significant improvements in both VO2 max and QoL according to the MLWHFQ. The data appeared to show a better response to treatment in the subgroup of patients with less severe systolic disfunction (an LVEF greater than 25%). There did not appear to be a correlation between the response to treatment and the etiology of heart failure [101]. Based on the results of the FIX-HF-5 study, a confirmatory trial was devised, which aimed at investigating the effects of CCM in a population of subjects with HF with an LVEF in the 25–45% range. The FIX-HF-5C study thus enrolled 160 HF patients, who were randomized to either OMT or OMT + CCM. Once again, the study design was unblinded, but the adjudication of events and the evaluation of the primary endpoint of VO2 was performed by a blinded laboratory. Similarly to the FIX-HF-5, the results showed a statistically significant improvement in VO2 max and QoL in the treatment group compared to the control, as well a considerably greater increase in 6MHWT distance, which increased on average by 43 m in the CCM group as opposed to 9.3 in the controls. Within the FIX-HF-5C population, the improvements observed in the treatment group were even more pronounced in the subgroup of patients which had an LVEF greater than 35. The number of deaths during the study was low in both groups; however, with this limitation, there was an improvement in survival free of cardiac death and HF hospitalization in the treatment group [102]. In a registry study of 143 patients with heart failure receiving CCM implantation, however, there appeared to be similar benefits between the two sub-populations with an EF greater or lower than 35%. Of note, in this case, the two populations had been accurately matched in terms of their symptoms and functional capacity, suggesting that the clinical picture may be relevant in predicting CCM responses [103]. In the trials discussed up to this point, the device under investigation was a three-lead system which employed an electrocatheter placed in the right atrial appendage to detect atrial activity. This information was used to allow for the correct timing of the CCM impulse during the absolute refractory period and to suppress stimulation over premature ventricular contractions. This configuration limits the use of the device in patients suffering from atrial fibrillation. A newer iteration of the device does away with the atrial lead and instead uses the two ventricular electrodes for both the sensing and delivery of the impulse. To test this device, 60 patients were enrolled in the FIX-HF-5C2 study. They were comparable to the FIX-HF-5C subjects and included patients with LVEF 25–45%, who were not candidates for CRT and had no revascularization planned. Patients who had a recent (<30 days prior) hospitalization or use of intravenous diuretics or inotropes were excluded. Crucially, 15% of the patients in this study had atrial fibrillation. The design of the FIX-HF-5C2 was to compare the subjects with those of the FIX-HF-5C. The results showed that the two-lead system was comparable to the three-lead in terms of the amount of stimulation given, while retaining a slightly improved safety profile [104]. Thus far, all the evidence discussed concerns patients with an LVEF in the reduced or mildly reduced range. As mentioned above, however, CCM appears to retain greater effectiveness in patients with less severe systolic disfunction (greater effectiveness in the 25–45% range of LVEF than in the overall population, and even more so in the 35–45% range) [105,106]. Thus, CCM represents an intriguing option for patients with a greater ejection fraction, particularly those with HFpEF, which constitute a group which has a remarkably narrow range of therapeutic options. A recently published pilot study enrolled a population of 47 patients with an LVEF greater than or equal to 50% and employed a single-arm design to show that CCM implantation afforded a clinically significant improvement in health status, as assessed via the Kansas City Cardiomiopathy questionnaire [107]. One further interesting development in CCM is the upcoming Optimizer Integra device, which will combine our capability to deliver CCM therapy with ICD functionality, thus allowing patients to undergo a single implantation procedure, as well as reducing the number of leads that need to be implanted.

## 8. Autonomic Modulation in HFrEF and HFpEF

Nearly half of all HF patients have HFpEF [6]. This population carries similar prognosis and hospitalization rates as those with HFrEF. Besides the positive results of (Aldosterone Antagonist Therapy for Adults With Heart Failure and Preserved Systolic Function) TOPCAT [108] and (Efficacy and Safety of LCZ696 Compared to Valsartan, on Morbidity and Mortality in Heart Failure Patients With Preserved Ejection Fraction) PARAGON-HF [109] with respect to the reduction of HF-related hospitalizations, Dapagliflozin and Empagliflozin are unique drugs to achieve a class I recommendation for HFpEF treatment [110]. The complex pathophysiology of HFpEF and its numerous etiologies may have contributed to the lack of effective therapies. Autonomic imbalance is thought to contribute to diastolic dysfunction through several mechanisms [111]. Renal denervation decreases the overall sympathetic tone of the heart by affecting sensory inputs and centrally modulating its autonomic tone. Renal denervation is particularly appealing for HFpEF, as this features multiple comorbidities such as hypertension, atrial fibrillation, and diabetes [112]. A recent retrospective study not only identified a high HFpEF prevalence in a population of patients with resistant hypertension who underwent renal denervation, but also significant hemodynamic improvements [113]. However, it is not clear whether these positive effects are a consequence of the normalization of blood pressure; therefore, further studies are needed both to gain mechanistic insights and to select the patients who are most likely to derive benefits from the intervention. Another device modulating autonomic disequilibrium and initially designed for the treatment of hypertension is the Barostim Neo. Typically, the baroreflex receives sensory information from receptors located in the aortic arch and carotid sinus, which are triggered by the arterial wall stretch. In turn, it modulates autonomic output, addressing variations in blood pressure [114]. The system comprises a pulse generator, which resembles in size and form a defibrillator, surgically placed in the pectoral area and a subcutaneous lead directed towards ipsilateral carotid bifurcation, providing baroceptor activation. A long-term analysis of the pivotal studies evaluating the device in resistant hypertension found greater benefits in symptomatic patients with signs of congestion and a preserved LVEF [115]. Earlier studies in symptomatic patients with an ejection fraction < 35% showed beneficial effects in terms of QoL and functional capacity alongside a significant reduction in NT-proBNP levels [116,117]. Unfortunately, the study’s design and inadequate statistical power did not allow for the evaluation of hard endpoints. Recently, long-term results of the BeAT-HF (Baroreflex Activation Therapy for Heart Failure, NCT02627196) study were published. BeAT-HF was a two-arm, parallel-group, open-label, non-implanted control trial. A total of 323 symptomatic HFrEF patients (NYHA ≥ III, EF ≤ 35%) were randomly assigned to GDMT alone or GDMT plus baroreflex activation therapy (BAT). Despite significant and sustained improvements in symptoms and functional class in the intervention group, primary composite endpoint (cardiovascular death and HF morbidity) event rates were not different between the two groups (RR 0.94, 95% CI 0.57–1.57; *p* = 0.82) [118]. A specific study of HFpEF patients with resistant hypertension is ongoing (BAROSTIM THERAPY In Heart Failure With Preserved Ejection Fraction (NCT02876042). Central sleep apnea (CSA) is a common feature of both HFrEF and HFpEF patients. CSA causes hypoxia and detrimental sympathetic activation with adverse cardiovascular effects [119]. Neurostimulation produces diaphragmatic contraction, emulating physiological breathing. On the one hand, traditional strategies have yielded disappointing results in patients with HF (i.e., CANPAP RCT) [120]. On the other hand, studies conducted with the Remedy system (Respicardia, Inc., Minnetonka, MN, USA) provided the first positive results of the remedé System Pivotal Trial (NCT01816776), which enrolled more than 50% participants with HF [121]. A post hoc analysis including 96 HF patients with an LVEF lower than 45% confirmed improvements in their QoL. Additionally, the study showed an increased LVEF with a non-significant trend towards lower left ventricular volumes and HF-related hospitalizations [122]. However, physicians need to be aware of potential interactions since inappropriate ICD shocks have been reported. 

## 9. Conclusions

Based on the effect of devices on the management of HFrEF, and backed by initial preclinical and clinical data on HFpEF, a device-based strategy shows significant potential in tackling a key current issue in cardiovascular medicine. The complex pathophysiology of HF, along with its wide range of risk factors, comorbidities, and symptoms, has posed significant challenges in the development of appropriate therapeutics. As novel device-based therapies are still being developed, clinical studies have mostly focused on evaluating device safety, mortality rates, and the occurrence of adverse events after implantation. As a result, there are still unresolved concerns about the safety and effectiveness of these devices in specific patient groups. Furthermore, prior to contemplating these devices, the traditional approach for treating HF involves initiating and optimizing GDMT. Indeed, this was a condictio sine qua non in RCTs evaluating devices in HF. This methodology relies on the chronological progression of devices that preceded the initial testing and authorization of medications for HF. The possibility of an early implementation of device-based therapy, prior or simultaneously to drug therapies, has not yet been investigated. Devices have several benefits compared to medications. They work mostly autonomously from the patient’s compliance. Furthermore, they focus on structural or biological processes that are usually not responsive to conventional pharmacological treatments. In addition, they often enhance hemodynamics without causing a decrease in blood pressure, heart rate, or renal function. As a result, their administration is not affected by changes in cardiovascular or kidney conditions. Conversely, improved hemodynamics could facilitate the optimization of medication treatments, as devices do not have any influence on medical therapy. Traditional methods significantly postpone the timely implementation of suitable treatments for eligible patients. The individualized sequencing of therapies, whether they include medications or devices, has the potential to expedite the realization of their beneficial effects. For example, in patients for whom tolerance is a barrier, a strategy that privileges device-based therapy prior to the achievement of GDMT may allow up-titration. Although it is reasonable to utilize devices in patients who cannot attain GDMT, there is an urgent need for studies specifically focused on investigating the order and fast escalation of all heart failure treatments, including medications and devices. This is necessary even for individuals who are considered to have a high tolerance for medications, as the residual risk remains significant.

## Figures and Tables

**Table 1:** Main studies evaluating mitral valve devices.

Trials	Device	Design	Population	Key Results
MITRA-FR [11,13]	Mitraclip	Randomized, controlled, open-label trial	304 HFrEF patients with severe MR (ERO 0.31 cm^2^, LV diameter 69 mm, NT-proBNP 3300 pg/mL)	*Death from any cause or unplanned HHF at 12 months* –Intervention Group: 54.6% –Control Group: 51.3% *Death from any cause or unplanned HHF at 24 months* –Intervention Group: 63.8% –Control Group: 67.1%
COAPT [12,18]	Mitraclip	Randomized, controlled, parallel-group, open-label trial	614 HF patients † with severe MR (ERO 0.41 cm^2^, LV diameter 62 mm, NT-proBNP 5500 pg/mL)	*HHF at 24 and 60 months, annualized rate* –Intervention Group: 35.8% *, 33.1% * –Control Group: 67.9%, 57.2% *Death from any cause at 24 and 60 months* –Intervention Group: 29.1% *, 57.3% * –Control Group: 46.1%, 67.2% *Change in KCCQ score at 12 months* –Intervention Group: 12.5 * –Control Group: −3.6 *Change in distance on 6-MWT* –Intervention Group: −2.2 m * –Control Group: −60.2 m
CLASP [19,20,22]	Pascal	Single-arm prospective study	109 symptomatic patients with severe MR ‡ (ERO 0.38 cm^2^, LV diameter 61 mm, NT-proBNP 4100 pg/mL)	*Baseline* vs. *30 days* –Survival: 98.4% –ERO: 0.38 vs. 0.17 cm^2^ * –Residual MR (≤1): 81% –6-MWT: 258 m vs. 295 m * –KCCQ Score: 55 vs. 71 * Baseline vs. *12 and 24 months* –Survival: 92%, 80% –ERO: 0.39 cm^2^ vs. 0.16 cm^2^ *, 0.22 cm^2^ * –Residual MR (≤1) 82%, 78% –Freedom from HHF: 87%, 84%
Cardioband With Transfemoral Delivery System [29]	Cardioband	Single-arm prospective study	60 HF patients with at least moderate MR	*Baseline vs. 12 months* –Survival: 87% –Freedom from HHF: 66% –Residual MR (≤2) 61% –MLHFQ: 20 vs. 39 * –6-MWT: 285 m vs. 342 m *
Mitralign Percutaneous Annuloplasty First in Man Study [25]	Mitralign	Single-arm prospective study	71 patients with at least moderate FMR and EF < 45%	*Baseline vs. 6 months* –Survival: 82% –ERO 0.33 cm^2^ vs. 0.29 cm^2^ –Residual MR (≤2) 52% –6-MWT: 307 m vs. 364 m *
REDUCE-FMR [26]	Carillon	Double-blinded, randomized, sham-controlled trial	120 patients with at least moderate FMR and EF < 50%	*Change in RV at 12 months* –Intervention Group: −7.1 * –Control Group: 3.3 *Change in 6-MWT at 12 months* –Intervention Group: 32 m –Control Group: 17.5 m *Change in KCCQ Score at 12 months* –Intervention Group: 9.5 –Control Group: 7.6
Expanded Clinical Study of the Tendyne Mitral Valve System [28]	Tendyne	Single-arm prospective study	100 patients with severe MR and high surgical risk	*Baseline* vs. *24 months* –Survival: 61% –Residual MR (≤1) 7% –6-MWT: 245 m vs. 287 m

*: *p* ≤ 0.05; †: 82% with EF < 40%; ‡: 65% with HF; EF: ejection fraction; HF: heart failure; HFrEF: Heart Failure with Reduced Ejection Fraction; ERO: effective regurgitant orifice; MR: mitral regurgitation; LV: left ventricle; NT-proBNP: N-terminal pro B-type Natriuretic Peptide; HHF: Heart Failure Hospitalization; KCCQ: Kansas City Cardiomyopathy Questionnaire; 6-MWT: 6 Minutes Walking Test; MLHFQ: Minnesota Living with Heart Failure Questionnaire; RV: regurgitant volume; MITRA FR: Multicentre Study of Percutaneous Mitral Valve Repair MitraClip Device in Patients with Severe Secondary Mitral Regurgitation; COAPT: Cardiovascular Outcomes Assessment of the MitraClip Percutaneous Therapy for Heart Failure Patients with Functional Mitral Regurgitation; CLASP: The CLASP Study Edwards PASCAL TrAnScatheter Mitral Valve RePair System Study; REDUCE-FMR: Carillon Mitral Contour System for Reducing Functional Mitral Regurgitation.

**Table 2:** Main studies evaluating tricuspid valve devices.

Trials	Device	Design	Population	Key Results
TRILUMINATE [32]	Triclip	Randomized controlled trial	350 patients with severe TR	*Primary hierarchical composite endpoint* † –Intervention Group vs. Control Group Win Ratio: 1.48 * Change in KCCQ Score at 12 months –Intervention Group: 12.3 * –Control Group: 0.6 *Change in 6-MWT at 12 months* –Intervention Group: −8 m –Control Group: −25 m *Residual TR (≤2)* –Intervention Group: 87% * –Control Group: 4%
CLASP-TR [33]	Pascal	Single-arm prospective study	65 patients with severe TR	*Baseline vs. 12 months* –Residual TR (≤2): 86% * –Freedom from All-Cause Death: 88% –Freedom from HHF: 78.5% –6-MWT: 208 m vs. 311 m * –KCCQ Score: 53 vs. 72 *
TRI-REPAIR [34]	Cardioband	Single-arm prospective study	30 patients with at least moderate TR	*Baseline vs. 12 months, 24 months* –Residual TR (≤2): 63% *, 72% * –6-MWT: 248 m vs. 296 m, 309 m –KCCQ Score: 45 vs. 64 *, 63 * –Freedom from All-Cause Death: 83%, 73% –Freedom from HHF: 69%, 56%
SCOUT [35]	Trialign	Single-arm prospective study	15 patients with at least FTR	*Baseline vs. 30 days* –TA: 12.3 vs. 11.3 * –ERO: 0.51 cm^2^ vs. 0.32 cm^2^ * –6-MWT: 245 m vs. 298 m * –MLHFQ: 47 vs. 21 *
Wild et al. [36]	Tricento	Retrospective observational registry	21 high-risk patients with at least severe TR	–RVEDV: 252 vs. 221 mm^3^ (median follow-up 188 days) –1-year survival rate: 76%
TRICUS-EURO [37]	Tricvalve	Single-arm prospective study	35 symptomatic patients (NYHA ≥ III) with at least severe TR	*Baseline vs. 1 month* –KCCQ Score: 42 vs. 59 –NYHA Class ≤ II: 50% *Baseline vs. 6 months* –KCCQ Score: 42 vs. 59 –NYHA Class ≤ II: 79%
TRISCEND [38]	Evoque	Single-arm prospective study	176 patients with at least moderate TR	*Baseline vs. 12 months* –Residual TR (≤1): 98% * –SV: 54 vs. 65 mL * –CO: 4 vs. 4.5 L/min * –KCCQ Score: 46 vs. 72 * –6-MWT: 214 vs. 270 *

*: *p* ≤ 0.05; † Death from any cause or tricuspid valve surgery, HHF, improvement in QoL as measured with the KCCQ; HHF: Heart Failure Hospitalization; QoL: quality of life; KCCQ: Kansas City Cardiomyopathy Questionnaire; TR: tricuspid regurgitation; 6-MWT: 6 Minutes Walking Test; FTR: Functional Tricuspid Regurgitation; TA: tricuspid annulus; ERO: effective regurgitant orifice; RVEDV: Right Ventricular End Diastolic Volume; NYHA: New York Heart Association; SV: Stroke Volume; CO: cardiac output; TRILUMINATE: TRILUMINATE Study with Abbott Transcatheter Clip Repair System in Patients with Moderate or Greater TR; CLASP-TR: Edwards PASCAL TrAnScatheter Valve RePair System in Tricuspid Regurgitation; TRI-REPAIR: TrIcuspid Regurgitation RePAIr with CaRdioband Transcatheter System; SCOUT: Early Feasibility of the Mitralign Percutaneous Tricuspid Valve Annuloplasty System (PTVAS) Also Known as TriAlign; TRICUS-EURO: Safety and Efficacy of the TricValveâ Transcatheter Bicaval Valves System in the Superior and Inferior Vena Cava in Patients With Severe Tricuspid Regurgitation.

**Table 3:** Main studies evaluating IASDs.

Trials	Device	Design	Population	Key Results
REDUCE LAP-HF I [54]	Corvia Atrial Shunt	Phase 2, randomized, parallel-group, blinded trial	44 patients with NYHA III, LVEF ≥ 40%, exercise PCWP ≥ 25 mmHg, and PCWP right atrial pressure gradient ≥ 5 mmHg	–Peak PWCP after 1 month: −3.5 mmHg * –No peri-procedural or 1-month MACCRE
REDUCE LAP-HF II [58]	Corvia Atrial Shunt	Randomized, blinded, sham-controlled trial	626 symptomatic patients with LVEF ≥ 40%, exercise PCWP ≥ 25 mmHg, and PCWP right atrial pressure gradient ≥ 5 mmHg	–No difference in primary composite endpoint –No differences in the composite safety endpoint
ALT-FLOW [61]	APTURE transcatheter shunt system	Single-arm open-label trial	87 symptomatic patients with exercise PCWP ≥ 25 mmHg, and PCWP right atrial pressure gradient ≥ 5 mmHg	*Baseline vs. 6 months* –KCCQ Score: 39 vs. 62 * –NYHA Class II: 12 vs. 67% * –20W PCWP: 35 vs. 28 mmHg
RELIEVE-HF [64]	V-Wave	Prospective, randomized, observer-blinded study	97 HF patients on GDMT with ≥1 HHF within 12 months	*Baseline vs. 12 months* –KCCQ Score: 46 vs. 59 * –LVEDVi: 77.7 vs. 74.4 ml/m^2^ * –LVESVi: 49 vs. 45.6 mL/m^2^ * –RVFAC: 36 vs. 40% * –TAPSE: 15.9 vs. 17.3 mm * –LVEF: 43 vs. 45% *

*: *p* ≤ 0.05; NYHA: New York Heart Association; LVEF: left ventricular ejection fraction; HF: heart failure; PCWP: pulmonary capillary wedge pressure; MACCRE: Major Adverse Cardiovascular Cerebrovascular Renal Event; KCCQ: Kansas City Cardiomyopathy Questionnaire; GDMT: guideline-directed medical therapy; LVEDVi: Left Ventricular End Diastolic Volume index; LVESVi: Left Ventricular End Systolic Volume index; RVFAC: Right Ventricular Fractional Area Change; TAPSE: Tricuspid Annular Plane Systolic Excursion; REDUCE LAP-HF I: Reduce Elevated Left Atrial Pressure in Patients With Heart Failure; REDUCE LAP-HF II: A Study to Evaluate the Corvia Medical, Inc. IASD System II to Reduce Elevated Left Atrial Pressure in Patients With Heart Failure; ALT-FLOW: Early Feasibility Study-Transcatheter Atrial Shunt System; RELIEVE: REducing Lung congestIon symptoms using the v-wavE shunt in adVancEd Heart Failure.

**Table 4:** Main studies evaluating CCM.

Trials	Device	Population	Key Results
FIX-HF-4 [100]	Three-lead Optimizer System	164 patients on stable GDMT for HFrEF, EF < 35%	Cross-over study, showed improvement in peak VO_2_ and MLWHFQ
FIX-HF-5 [101]	Three-lead Optimizer System	428 patients with advanced heart failure (EF less than 35% and NYHA class III or IV)	Unblinded prospective study, showed improvements in peak VO_2_ and MLHFQ and suggested greater effect in the subgroup with higher EFs
FIX-HF-5C [102]	Three-lead Optimizer System	160 patients with HF with an EF in the 25–45% range	Confirmed greater efficacy in patients with an EF > 35%
FIX-HF-5C2 [104]	Two-lead Optimizer Smart System	60 patients with HF with an EF in the 25–45% range (15% of whom suffered from atrial fibrillation)	Showed that there is no sacrifice in efficacy with the two-lead Optimizer device
CCM-Reg [106]	Two-lead Optimizer Smart System	140 patients, EF in the 25–45% range	Improved QoL and NYHA class, reduced hospitalizations, survival better than predicted
CCM-HFpEF [107]	Two-lead Optimizer Smart System	47 patients with an EF > 50%	Improved QoL according to KCCQ, small improvements in echo parameters

GDMT: guideline-directed medical therapy; EF: ejection fraction; HFrEF: Heart Failure with Reduced Ejection Fraction; VO_2_: Oxygen Uptake; MLHFQ: Minnesota Living with Heart Failure Questionnaire; NYHA: New York Heart Association; QoL: Quality of Life; KCCQ: Kansas City Cardiomyopathy Questionnaire; FIX-HF-5: Evaluate Safety and Efficacy of the OPTIMIZER^®^ System in Subjects With Moderate-to-Severe Heart Failure; FIX-HF-5C2: Evaluation of the Safety and Efficacy of the 2-lead OPTIMIZER^®^ Smart System; CCM-Reg: CCM Registry; CCM-HFpEF: CCM in Heart Failure With Preserved Ejection Fraction.

## Data Availability

Not applicable.
